# Reply to “The Fallacy of Using Administrative Data in Assessing the Effectiveness of Food Fortification. Comment on: Folic Acid Fortification and Neural Tube Defect Risk: Analysis of the Food Fortification Initiative Dataset. *Nutrients* 2020, *12*, 247”

**DOI:** 10.3390/nu12051335

**Published:** 2020-05-08

**Authors:** Cara J. Westmark, Michaela E. Murphy

**Affiliations:** 1 Department of Neurology, University of Wisconsin-Madison, Madison, WI 53706, USA; 2 Nutritional Sciences, University of Wisconsin-Madison, Madison, WI 53706, USA; memurphy6@wisc.edu

## 1. Introduction

We would like to thank Kancherla et al. for carefully reading and commenting [1], on our recent publication, “*Folic Acid Fortification and Neural Tube Defect Risk: Analysis of the Food Fortification Initiative Dataset*” [2]. Kancherla et al. disagree with our statistical analysis and conclusions. We contend that our methods were sound and that we provided a fair and balanced analysis of the evidence-based literature regarding the pros and cons of national folic acid supplementation. We thank the editors of Nutrients for the opportunity to respond to their criticisms in detail. Food fortification is a controversial issue of immense public health importance, and we appreciate the opportunity to highlight the need for further research.

In 1992, based on observational studies, the USA Public Health Service (PHS) recommended that all women of childbearing age consume 400 μg of folic acid daily to prevent neural tube defects (NTD) in pregnancy [3]. To promote compliance, the USA was the first country to mandate a national food fortification program, requiring that all enriched cereal grain products be fortified with folic acid. Currently, more than 80 other countries fortify cereal grains with folic acid. Over the past three decades, concerns have been raised that folic acid supplementation may negatively impact certain subpopulations—for example, the elderly [4].

The main objective of our paper [2] was to retrospectively address the question of whether folic acid fortification improved NTD at the population level. Toward that goal, we extracted and analyzed relevant data from the Food Fortification Initiative (FFI) dataset. Our study compared countries with national folic acid fortification versus countries without national folic acid fortification, with the rationale that a national fortification policy would directly affect the vast majority of inhabitants of a particular country, despite the importation of grain products from other countries. The primary endpoint of interest examined was the prevalence of NTD. Our overall finding was that “*national fortification with folic acid is not associated with a significant decrease in the prevalence of neural tube defects at the population level*”.

## 2. Ecological & Exception Fallacies

Kancherla et al. assert that we “*made an error of ecological fallacy”* in our conclusion. Ecological fallacy occurs when an analysis of group data is used to draw conclusions about an individual. A classic example is a study showing that people who wear eyeglasses have a higher than average IQ level, and then concluding that an individual who wears eyeglasses has a higher than average IQ. By definition, we did not commit “*ecological fallacy*”, as both our data analysis and our conclusion were made at the population level.

Ironically, a strong case can be made that proponents of national food fortification with folic acid have committed an “*exception fallacy*”. Exception fallacy is the reversal of ecological fallacy and occurs when data about individual cases is used to draw conclusions about a group of people. There is substantial evidence that inadequate folate levels can cause NTD; however, this does not mean that all pregnant women will benefit from folic acid supplementation or that the majority of the general population who are not and cannot become pregnant will benefit from folic acid fortification. Moreover, it is not clear to what extent national folic acid supplementation benefits pregnant women who are deficient in folate, as we do not have a national monitoring program, do not track consumption at the individual level, and do not know the benefits and risks to individuals with specific genetic mutations. Even if data were available that showed a moderate or strong correlation between improved NTD outcomes and folic acid supplementation at the population level, with consideration of relevant confounding issues, a strong argument could be made that the total available evidence does not support national supplementation with folic acid because numerous subgroups in the population are potentially harmed. Folic acid can reduce the blood levels of certain medications and increase the risk of certain types of cancer.

## 3. Confounding Issues Associated with the Study

Population-level observational (ecological) studies are not without numerous potentially confounding issues. Our study is unique in addressing the confounding issue of the lack of control groups in observational folic acid fortification studies. The average prevalence of NTD per 10,000 births in countries that do not fortify any cereal grains with folic acid was 13.32 (SD: 5.50, *n* = 116 countries), and the average prevalence of NTD in countries with at least one cereal grain fortified with folic acid was 13.30 (SD: 6.13, *n* = 70). The range of prevalence of NTD per 10,000 births was similar with fortification (5–32 NTD per 10,000 births) and without fortification (4–32 NTDs per 10,000 births). We acknowledged several potential confounding issues in our manuscript, including variations in consumption of folic acid, genetic variants and overall nutritional status at the individual subject level, the accuracy of data reporting agencies, voluntary fortification with folic acid, periconception folic acid supplementation, and the variable implementation timing of mandatory folic acid programs. These confounding issues could not be addressed with the available data.

**Figure 1:**
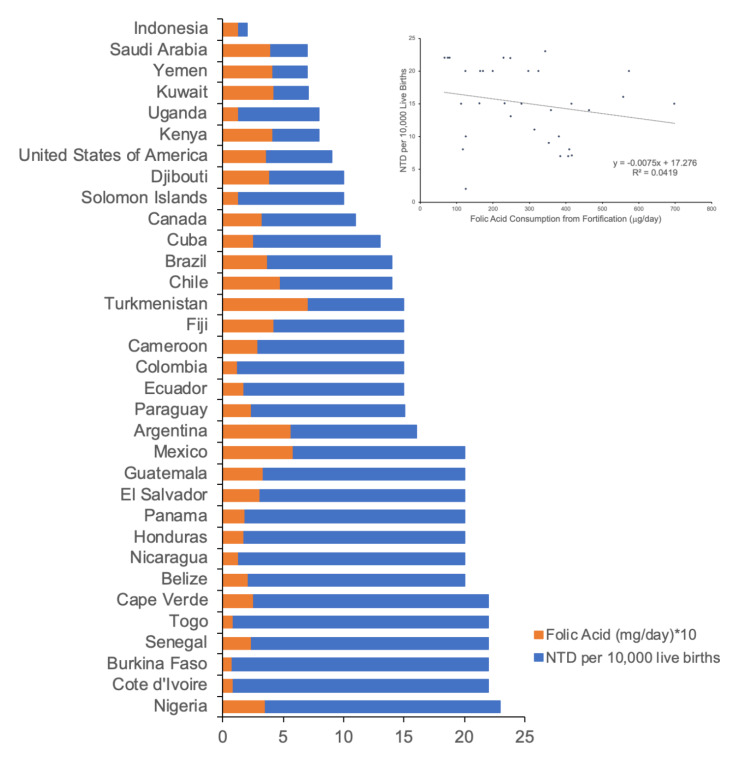
Prevalence of neural tube defects (NTD) (spina bifida and anencephaly) as a function of folic acid consumed from fortification. The number of NTD per 10,000 live births were plotted (blue bars) versus country (*n* = 33). Folic acid consumption levels from fortification in mg/day*10 (orange bars) were superimposed on NTD prevalence. A linear regression analysis indicates a regression coefficient of −0.0075.

Kancherla et al. list additional confounding issues of, “incorrect considerations on NTD prevalence, average grain availability for a country, fortification coverage in a county, population reach of fortified foods within country, absence of consideration of fortification type (voluntary vs. mandatory), country-specific policies on elective terminations for NTD-affected pregnancies, stillbirth proportions among those with NTDs, and fortification implementation”. In response, we agree with Kancherla et al. that there are numerous potentially confounding issues in analyzing the efficacy of national food fortification with folic acid. Herein, we address the issues of NTD prevalence, average grain availability for a country, fortification coverage in a county, the population reach of fortified foods within country, and the absence of the consideration of fortification type (voluntary vs. mandatory) by conducting a secondary analysis of Kancherla et al.’s dataset [5] at the country level. In their paper, entitled “A 2017 global update on folic acid-preventable spina bifida and anencephaly”, Kancherla et al. extracted data from the FFI dataset regarding country-specific levels of folic acid fortification in ppm (the same as we did), as well as estimated the daily intake of total folic acid from fortified cereal grains and calculated the fortification program coverage. Using their reported annual number of live births, annual number of births with spina bifida and anencephaly, calculation of total folic acid consumption from fortification in μg/day, and calculation of program coverage, there were data available for 59 countries, with a fortification program coverage range of 0–100%. Extracting the data for 91% coverage (level of the USA) and greater, data were available for 33 countries (Figure 1). A linear regression analysis indicated a very weak correlation between NTD prevalence and the level of consumed folic acid from fortification (regression coefficient = −0.0075), which was similar to our reported results. Likewise, an analysis of the 25 countries with 100% fortification program coverage gives a regression coefficient of −0.0081 (data not shown). 

Overall, we utilized the most comprehensive and complete dataset available for our analysis, which is the same dataset used to promote mandatory large-scale folic acid fortification of staple grains. It is not possible to address all of the potentially confounding issues with the available data. Regardless, the remaining confounding issues apply to both our study and studies promoting national food fortification with folic acid and, to our knowledge, these issues have not been assessed in either context. 

## 4. Literature

Kancherla et al. claim that our study conclusion, “*contradicts several systematic reviews and meta-analyses published earlier*”, citing four papers [6,7,8,9]. Our paper was not meant to be a comprehensive systematic review of the literature. Nonetheless, we believe we presented a fair and balanced overview of the evidence-based literature regarding the national supplementation of cereal grains with folic acid, including both the pros and cons. In our manuscript, we cite and discuss numerous individual studies, as well as conducting a systematic review and meta-analysis describing a lower prevalence of spina bifida in response to folic acid fortification. We also discuss two important confounding issues with these studies, in that they do not take into account declining NTD rates prior to folic acid fortification and they do not include comparisons to non-fortification control groups during the same time period. There are many reasons why NTD could decrease over time, irrespective of folic acid fortification—for example, improved health care or socioeconomic (SES) conditions. In the prior section, we discuss how we addressed the confounding issue of the lack of control groups in observational folic acid fortification studies. 

The three papers cited by Kancherla et al. that were not cited in our paper were systematic reviews and meta-analyses [6,7,9]. Specifically, these analyses showed a substantial decrease in NTD in Latin American countries, particularly in Chile and Costa Rica, after the fortification of cereal grains with folic acid. It is commendable that attempts were made to assess pre-fortification time trends in NTD prevalence by monitoring the congenital malformations reported in hospital records. However, none of these studies took into account changing SES conditions concurrent with the implementation of mandatory national folic acid policies in Latin America. For example, Chile started to rebuild its political system in 1990 from a military to a democratic-based government, which resulted in a higher expenditure on social programs to tackle poverty and poor-quality housing. These improved SES conditions were concurrent with the folic acid fortification of grains. Chile currently has the highest nominal gross domestic product (GDP) per capita in Latin America. Costa Rica also has one of the highest standards of living in Latin America, as their economy has evolved from a solely agricultural one to one based on tourism, electronics and the export of medical components.

In our paper, we assessed NTD prevalence in response to SES. We found a strong linear relationship between reduced NTD and increased GDP spent on SES indicators. Our findings suggest that improved NTD outcomes are associated far less, if at all, with mandatory folic acid fortification at the population level than with SES, as indicated by a greater than 30% reduced prevalence of NTD between the lowest and highest SES quintiles [2]. It remains to be determined if improved NTD outcomes, as a function of SES, are due to periconception folic acid supplementation with prenatal vitamins.

The Cochrane Database of Systematic Reviews, the leading journal and database for systematic reviews in health care, published a systematic review assessing the efficacy of folic acid fortification on health outcomes in the overall population and concluded that the evidence level was “*low certainty*” regarding the efficacy of folic acid fortification in improving NTD outcomes [10].

## 5. Response to Major Limitations Cited by Kancherla et al.

In response to the major limitations of our study listed by Kancherla et al. [1] we cite their assessment of the limitation in quotes, followed by our response.

(1) “*The modeled prevalence estimates for neural tube defects used in their analysis have inherent biases and limitations, and they underestimate the true prevalence of NTDs for many developing countries that lack birth defect surveillance. They are mainly intended to provide policy makers with a crude burden of NTDs and not for scientific hypothesis-oriented research*”. We utilized NTD prevalence estimates reported by the FFI, who cite Blencowe et al. [11] for the majority of their estimates. In Figure 1, we obtain similar results using NTD prevalence estimates from Kancherla et al. [5]. These are the only publicly available estimates for many countries and are the data used to promote the national fortification of cereal grains;
(2) “*FFI’s individual country profiles that contain grain fortification-related information are intended for stakeholders in the flour and milling industry, and organizations and policy-makers invested in grain fortification. Their variable, “Folic acid fortification measured in ppm” is an incomplete measure of fortification reach and impact. The average fortification levels are meaningless without integrating data on the average grain availability for a country. A low fortification level in a country with high grain availability, would have a very different fortification impact compared to a low fortification level in a country with low grain availability*”. On the surface, this argument is reasonable in that the average grain availability for a country will affect the impact of fortification. There will be countries with high grain availability and others with low grain availability. We agree that our variable of folic acid fortification measured in ppm does not take into account reach and impact. This was encompassed in our discussion of the confounding issues related to variations in consumption of folic acid, the accuracy of data reporting agencies, voluntary fortification with folic acid, periconception folic acid supplementation, and variable implementation timing of mandatory folic acid programs. However, our analysis showed an equivalent average as well as a range of high and low values for NTD per 10,000 births with and without fortification. One would expect that if national fortification was working, then there should be at least a trend toward less NTD in the fortification cohort compared to no fortification. In addition, using fortification program coverage data generated by Kancherla et al. [5], a linear regression analysis indicated a very weak correlation between NTD prevalence and the level of folic acid consumed from fortification (Figure 1), which was similar to our reported result;
(3) “*Fortification coverage in a country was not considered available online …. Several countries have a mandatory fortification policy, but where fewer than 100% individuals consume fortified food*”. Please see Figure 1 and the associated discussion. The consideration of fortification coverage at both 100% and 91% or greater, with coverage rates reported by Kancherla et al. [5], did not change the results. Even with mandatory fortification, it is highly unlikely that 100% of individuals consume fortified cereal grains. For example, individuals with Celiac disease do not consume wheat;
(4) “*Population reach of fortified foods, which indicates actual consumption of fortified foods, was not considered. Analyzing population averages fortification levels (ppm), without considering the reach and coverage of the fortified product, masks differences found between consumers and non-consumers*”. We agree that population-level observational studies of food fortification mask differences between consumers and non-consumers. Optimal research would include a combination of population- and individual-level studies. Data is not available at the individual level and it is at the individual level that folic acid may interact with medications, genetic variations or other factors to help or harm health;
(5) “*Other reviews showing effectiveness of fortification on NTD prevention were not cited*”. We address this criticism above in the Literature section;
(6) “*Several supporting factors including fortification type (voluntary vs. mandatory), country-specific policies on elective termination for NTD-affected pregnancies, stillbirth proportions among those with NTDs, fortification implementation and coverage were not considered*”. We address the mandatory fortification and coverage criticisms in Figure 1. Regarding the abortion rates of NTD-affected pregnancies, of the top 10 countries with the highest number of abortions (Greenland, Russia, Hungary, Cuba, Nagarno-Karabakh, Czech Republic, Estonia, Martinique, Bulgaria, and China), NTD estimates per 10,000 births are available from the FFI for seven countries (Russia, seven; Hungary, 10; Cuba, eight; Czech Republic, 10; Estonia, nine; Bulgaria, 30; China, 19). In this group, Cuba is the only country with mandatory fortification of folic acid. The Kancherla et al. data report 13 cases of NTD per 10,000 live births in Cuba [5]. With this small dataset, NTD estimates range from 7–30, with an average of 14.17 per 10,000 births in countries that do not have mandatory fortification, but do have a high number of abortions, suggesting that the elective termination of NTD-affected pregnancies is likely not a confounding factor in the population analysis.

## 6. Conclusions

In conclusion, we did not find a reduced prevalence of NTD at the population level in response to national folic acid fortification, but rather the stratification of the data based on SES indicated a strong linear relationship between reduced NTD and better SES. In our opinion, there is a need to reconsider our national food fortification policy in regard to folic acid. We commend the efforts of public health scientists working to find a safe and inexpensive means to reduce NTD. However, in the absence of prospective monitoring of fortification programs, it is not possible to establish a cause and effect relationship between the risks and benefits of national folic acid fortification. Actual exposure levels and downstream effects are unknown. Mandatory folic acid fortification in the USA was projected to increase the average folic acid intake by 100 μg/day; however, the mean increase was approximately twice as large as projected [12]. The prevalence of individuals that exceed the upper limit for folic acid intake is 10% in the subset of the USA population that consumes folic acid supplements [13]. There are several vulnerable populations that may be adversely affected by folic acid, such as the elderly who have low vitamin B12 levels, those taking medications such as proton pump inhibitors, and those with certain methylenetetrahydrofolate reductase (MTHFR) polymorphisms. As a nation, we would not fortify our food with drugs like metformin or insulin to treat a large percentage of the population that has type 2 diabetes, because we would not be able to control the dose, because there are potential drug–drug interactions with other medications, and because it could lead to potential harm for many. For the same reasons, it is our opinion that we need a targeted approach to folic acid supplementation to prevent NTD in pregnancy. All women do not receive prenatal care. One targeted approach would be the national promotion of vitamin supplements containing methyl folate for women of childbearing age.

On a final note, we did not commence this research with an agenda or an a priori hypothesis. We were simply interested in determining the strength of the evidence-based literature regarding national folic acid supplementation. Both authors have a family history of Celiac disease, which excludes the consumption of fortified cereal grains and could contribute to deficient vitamin levels. Adequate folate intake is extremely important and supplementation with synthetic folic acid may benefit certain individuals; however, considering the known risks in exceeding the upper tolerable limit, potential harm to known subpopulations, the lack of monitoring outcomes, and the lack of dose control, we conclude that the evidence is weak at best to support current national supplementation policies. We hope that our findings, in conjunction with the Comment by Kancherla et al., and this reply continues a collegial debate on the strengths and weaknesses of the evidence-based literature regarding the national folic acid supplementation of cereal grains and promotes further research on this important topic of public health relevance.
